# Long-term exposure to muscarinic agonists decreases expression of contractile proteins and responsiveness of rabbit tracheal smooth muscle cells

**DOI:** 10.1186/1471-2466-14-39

**Published:** 2014-03-10

**Authors:** Rodopi Stamatiou, Efrosini Paraskeva, Anna Vasilaki, Ilias Mylonis, Paschalis Adam Molyvdas, Konstantinos Gourgoulianis, Apostolia Hatziefthimiou

**Affiliations:** 1 Laboratory of Physiology, Department of Medicine, School of Health Sciences, University of Thessaly, 3 Panepistimiou Str, 41500 BIOPOLIS Larissa, Greece; 2 Laboratory of Pharmacology, Department of Medicine, School of Health Sciences, University of Thessaly, 3 Panepistimiou Str, 41500 BIOPOLIS Larissa, Greece; 3 Laboratory of Biochemistry, Department of Medicine, School of Health Sciences, University of Thessaly, 3 Panepistimiou Str, 41500 BIOPOLIS Larissa, Greece; 4 Department of Respiratory Medicine, Department of Medicine, School of Health Sciences, University of Thessaly, 3 Panepistimiou Str, 41500 BIOPOLIS Larissa, Greece

**Keywords:** Airway smooth muscle, Acetylcholine, Carbachol, Phenotype, Proliferation

## Abstract

**Background:**

Chronic airway diseases, like asthma or COPD, are characterized by excessive acetylcholine release and airway remodeling. The aim of this study was to investigate the long-term effect of muscarinic agonists on the phenotype and proliferation of rabbit tracheal airway smooth muscle cells (ASMCs).

**Methods:**

ASMCs were serum starved before treatment with muscarinic agonists. Cell phenotype was studied by optical microscopy and indirect immunofluorescence, using smooth muscle α-actin, desmin and SM-Myosin Heavy Chain (SM-MHC) antibodies. [*N*-methyl-^3^H]scopolamine binding studies were performed in order to assess M_3_ muscarinic receptor expression on isolated cell membranes. Contractility studies were performed on isolated ASMCs treated with muscarinic agonists. Proliferation was estimated using methyl-[^3^H]thymidine incorporation, MTT or cell counting methods. Involvement of PI3K and MAPK signalling pathways was studied by cell incubation with the pathway inhibitors LY294002 and PD98059 respectively.

**Results:**

Prolonged culture of ASMCs with acetylcholine, carbachol or FBS, reduced the expression of α-actin, desmin and SM-MHC compared to cells cultured in serum free medium. Treatment of ASMCs with muscarinic agonists for 3-15 days decreased muscarinic receptor expression and their responsiveness to muscarinic stimulation. Acetylcholine and carbachol induced DNA synthesis and increased cell number, of ASMCs that had acquired a contractile phenotype by 7 day serum starvation. This effect was mediated via a PI3K and MAPK dependent mechanism.

**Conclusions:**

Prolonged exposure of rabbit ASMCs to muscarinic agonists decreases the expression of smooth muscle specific marker proteins, down-regulates muscarinic receptors and decreases ASMC contractile responsiveness. Muscarinic agonists are mitogenic, via the PI3K and MAPK signalling pathways.

## Background

The airway smooth muscle is implicated in the pathological process of chronic airway diseases, such as asthma and chronic obstructive pulmonary disease [1]. These diseases exhibit common features like increased parasympathetic activity and acetylcholine release [2], or airway remodeling [3]. Airway remodeling comprises changes in the composition, quantity and organization of airway wall components as consequence of chronic injury and repair of the airway epithelial–mesenchymal trophic unit [1,4]. The increased thickness of the smooth muscle layer could enhance shortening, leading to increased airway narrowing and airflow obstruction [1].

ASMC cultures are a mixed population of cells exhibiting variability between contractile and synthetic-proliferative phenotypes [5]. The distinction between ASMC phenotypes is based on the different expression of proteins implicated in the contraction mechanism, proliferation ability and protein synthesis. Synthetic-proliferative ASMCs appear to proliferate, while the features of contractile ASMCs are similar to those of the cells normally present in intact airway tissue [4]. Different stimuli drive the transition between the different ASMC phenotypes. Although the mechanism involved is not yet well understood, evidence supports the hypothesis that the p42/p44 MAPK pathway is involved in the shift of ASMCs toward a less contractile phenotype [6-9]. On the other hand, serum deprivation increases the percentage of cells that exhibit the “contractile phenotype” in ASMC culture [10]. The shift of ASMCs from synthetic-proliferative to contractile phenotypes is attended by a decrease in M_2_ and a parallel increase in M_3_ expression [11].

ASMCs express abundant G_i_ coupled muscarinic M_2_ and G_q_ coupled M_3_ muscarinic receptors [12,13], stimulation of which leads to the activation of the MAPK and the PI3K signalling pathways [14]. M_2_ and/or M_3_-receptor stimulation by muscarinic agonists could affect ASMC proliferation, but until now muscarinic receptor agonists have been reported to be mitogenic for ASMCs mainly in combination with growth factors [11,15], acting through M_3_ muscarinic receptors [11]. On the other hand, the effect of muscarinic agonists on ASMC phenotype and in consequence their ability to proliferate has not been thus far fully addressed. In the present study we investigate the effect of prolonged cell incubation with muscarinic agonists, acetylcholine or carbachol, on phenotype shifting and cell contractility of ASMC obtained from rabbit trachea. We also studied the mitogenic effect of muscarinic agonists on ASMC in relevance with their proliferative or contractile phenotype.

## Methods

### Animals

Adult rabbits were maintained in individual cages in a controlled environment and were provided with food and water before use for the study. Animals were treated in compliance with ethical and institutional guidelines. All protocols were approved by the Ethics Committee of the Department of Medicine, School of Health Sciences of University of Thessaly. Rabbits were euthanised by an overdose of intravenously administrated Pentothal (Abbott, Abbott Park, IL, USA).

### ASMC isolation and culture

The isolation of ASMC from adult rabbit trachea was done as previously described [16]. Briefly, tracheal smooth muscle was epithelium denuded, dissected from cartilage and washed in low Ca^2+^ Krebs solution (139 mM NaCl, 5.4 mM KCl, 1.47 mM MgSO_4_, 11 mM glucose, 1.47 mM KH_2_PO_4_, 2.8 mM Na_2_HPO_4_, 1.4 mM NaHCO_3_, 0.2 mM CaCl_2_). Tracheal smooth muscle was digested in 2 ml of low Ca^2+^ Krebs solution containing 0.25% bovine serum albumin (BSA), 2 mg/ml collagenase I and 10 U/ml elastase IV, for 30 min at 37°C with vigorous shaking. Then it was washed in low Ca^2+^ Krebs solution, centrifuged (1000 rpm for 10 min) and incubated for 45 min in low Ca^2+^ Krebs solution containing 0.25% BSA, 1 mg/ml collagenase I and 20 U/ml elastase IV. Dispersed ASMCs were washed and centrifuged (1000 rpm for 10 min) twice in Dulbecco’s modified Eagle’s medium/Ham/F12 (DMEM/F12) containing 10% fetal bovine serum (FBS), 100 U/ml penicillin and 100 g/ml streptomycin. The isolated ASMC were placed in culture flasks and grown at 37°C in a humified incubator under 5% CO_2_.

### Study design

Isolated tracheal ASMCs were serum starved by incubation in Dulbecco’s modified Eagle’s medium/Ham/F12 (DMEM/F12) containing 100 U/ml penicillin and 100 g/ml streptomycin, as indicated, and treated with the muscarinic agonists, acetylcholine (ACh; 10^-7^ M-10^-3^ M), or carbachol (CCh; 10^-9^ M-10^-5^ M). The effect of the muscarinic agonists on the ASMC phenotype was studied using optical microscopy, as well as indirect immunofluorescence, and Western blot analysis with anti-smooth muscle α-actin mouse monoclonal antibody, anti-myosin (smooth) clone HSM-V mouse monoclonal antibody and anti-desmin rabbit polyclonal antibody. The density of muscarinic receptors on ASMCs was studied with [*N*-methyl-^3^H]scopolamine binding on isolated cell membranes and the effect of acetylcholine and/or carbachol on the contractile ability of individual ASMCs in culture was estimated by counting the cell area before and after muscarinic agonist treatment in the presence or absence of atropine.

Cell proliferation was estimated using the methyl-[^3^H]thymidine incorporation, the MTT-Cell Titer 96® AQueous One Solution Assay (Promega) and Trypan blue cell counting methods. In the MTT cell proliferation assay, the optical density (OD) of cells incubated in serum-free medium (24 h) was used as control (set at 100%). Results are presented as percentage of OD of the control cells, while the DNA synthesis is presented as cpm of radioactive material incorporated in ASMCs. The possible involvement of PI3K and MAPK signalling pathways was studied using the pathways inhibitors LY294002 and PD98059 respectively.

### Cell culture treatment

ASMCs were trypsinised, counted and seeded into appropriate cell culture plates. They were allowed to adhere overnight, washed twice with phosphate buffered saline (1XPBS) and pre-incubated in FBS-free DMEM/F12 medium, containing 100U/ml penicillin and 100 μg/ml streptomycin. ASMCs were incubated in serum free medium for 1 or 7 days, and incubated in 10% FBS-containing medium or serum free medium in the presence or absence of increasing concentrations of the muscarinic agonists; acetylcholine (10^-7^ M-10^-3^ M) and carbachol (10^-9^ M-10^-5^ M) as indicated. Specifically, cells were incubated with ACh (10^-7^ M) or CCh (10^-9^ M) in morphology observation and thymidine incorporation experiments, with ACh (10^-5^ M) or CCh (3 × 10^-7^ M) in contractile phenotype marker protein expression, M_3_ muscarinic receptor expression and cell proliferation experiments and with ACh (10^-3^ M) or CCh (10^-5^ M) in responsiveness experiments. The culture medium was replaced every 3 days, during the incubation periods. For the study of the possible involvement of PI3K and/or MAPK signalling pathways, LY294002 (20 μΜ) and PD98059 (100 μΜ) inhibitors were added in cell culture medium 15 or 60 min prior to the addition of the muscarinic agonists, respectively.

### Cell morphology

Cell morphology was observed with a reverse microscope Nikon Diaphot 300 (Nikon Inc., Melville, NY, USA).

### Indirect immunofluorescence

Cells were plated onto glass slides, which were resided in culture plates and treated as indicated above. After the treatment, cells were fixed with PBS-3% formaldehyde and permeabilized with PBS-1% Triton X-100, blocked in PBS-0.1% Tween 20- 3% BSA and incubated with anti-smooth muscle α-actin mouse monoclonal antibody (1:1000, Sigma), anti-myosin (smooth) clone HSM-V mouse monoclonal antibody (1:1000, Sigma) and anti-desmin rabbit polyclonal antibody (1:1000, Sigma), followed by incubation with an anti-mouse IgG or anti-rabbit IgG antibodies conjugated with CY3. Finally, coverslips were mounted on Vectrashield solution containing DAPI for DNA staining and the percentage of the cells that expressed α-actin, SM-MHC (Smooth muscle-Myosin Heavy Chain) and desmin proteins was estimated.

### Western blot analysis

Cells were lysed in 20 mM Tris-Cl pH 8.0, 150 mM NaCl, 1% Triton X-100, 1 mM dithiothreitol, and 100 mg/mL-1 phenylmethylsulphonyl fluoride. Total cell extracts were cleared by centrifugation (10000 g for 20 min at 4°C). 40 mg of protein were analysed in 10% sodium dodecyl sulphate–polyacrylamide electrophoresis gel and transferred to a nitrocellulose membrane. Western blot analysis was performed using anti- smooth muscle β-actin mouse monoclonal antibody (1:5000, Cell Signalling), anti-smooth muscle α-actin mouse monoclonal antibody (1:1000, Sigma), anti- myosin (smooth) clone HSM-V mouse monoclonal antibody (1:1000, Sigma) and anti-desmin rabbit polyclonal antibody (1:1000, Sigma). Membranes were then incubated with horseradish peroxidase conjugated anti-rabbit IgG (1:3000, Cell Signalling) or anti-mouse IgG (1:3000, Cell Signalling), and signals visualised by enhanced chemoluminescence (ECL).

### [*N*-methyl-^3^H]scopolamine binding studies

The binding of [*N*-methyl-^3^H]scopolamine ([^3^H]NMS) was done as previously described [17]. Briefly, ASMCs were serum deprived for 24 h and then incubated for 3, 7 or 15 days with 10% FBS, ACh (10^-5^ M) or CCh (3 × 10^-7^ M). Control cells were incubated in serum free medium. Cells were lysed by scraping in buffer (25 mM Tris-HCl, 2.5 mM CaCl_2_, 1 mM PMSF, 1 mM Pefabloc and 10 μg/ml aprotinin). Cell extracts were homogenized with 5 strokes in a Heidolph Silent Crusher S (Heidolph Instruments GmbH & Co. KG, Germany) and the homogenate was centrifuged at 10000 g for 5 min at 4°C. The supernatant was diluted up to 11 ml in assay buffer (10 mM HEPES, 100 mM NaCl, 10 mM MgCl_2_) and centrifuged at 150000 g for 30 min at 4°C. The pellet was then resuspended in assay buffer and subjected to sonication for 5 sec. 30 μg of protein was added in the reaction, along with 5 nM of [^3^H]NMS. Nonspecific binding was estimated by the presence of 100 μM atropine. After 80 min of incubation in order to allow radioligand binding only to M_3_ receptors [17], the samples were filtered through GF/B filters (Millipore) using a Millipore vacuum filtration system (EMD Millipore Corporation, Billerica, MA, USA). Before radiation counting in a Wallac β-counter, filters were dried overnight and incubated in Ultimate gold XR (Perkin Elmer) scintillation fluid for 1 h at RT.

### Contractility studies

ASMCs were grown to confluence in culture plates and incubated 3-15 days in serum-free medium, or medium containing 10% FBS, acetylcholine (10^-5^ M), carbachol (3 × 10^-7^ M). The cells were washed twice with phosphate buffered saline (1xPBS), and then incubated for 5 min with trypsin-EDTA solution, in order to detach myocytes from the substrata. The detached cells were incubated with acetylcholine (10^-3^ M) or carbachol (10^-5^ M) for 5 min. Photographs were taken before and after cell treatment, in a phase-contrast microscopy Nikon Diaphot 300 (Nikon Inc., Melville, NY, USA) with Leica DFC480 Camera (Leica Microsystems, Wetzlar, Germany). The ratio of total cell area before and after treatment was measured using the Image J programme. To confirm that contractile shortening responses were the direct result of ACh or CCh stimulation, atropine (10^-6^ M) was added in control experiments before ACh or CCh exposure.

### Cell proliferation studies

Proliferation of cultured ASMCs was estimated using (A) the methyl-[^3^H]thymidine incorporation method,(B) the MTT-Cell Titer 96® AQueous One Solution Assay (Promega) method and (C) the Trypan blue method.

(A) DNA synthesis in airway smooth muscle cells was estimated by measuring methyl-[^3^H]thymidine incorporation. Methyl-[^3^H]thymidine was added in the culture medium the last 18 h of incubation. The counts per minute (cpm) of the radioactive DNA were counted using a Wallac scintillation counter. Cells incubated in serum free medium were used as negative control in each experiment. The proliferating capability of cells was evaluated by positive controls, which were cells incubated in 10% FBS.

(B) The number of airway smooth muscle cells was estimated using the Cell Titer 96® AQueous One Solution Assay (Promega) method as previously described [18]. The absorbance of the MTT formazan reduction product was measured at 490 nm with a reference at 630 nm in an ELISA plate reader. The measured optical density (OD) is reminiscent of the cell number in the well, since there is a linear response between the measured OD and cell number (data not shown).

(C) Cell number was estimated by direct cell counting, using the Trypan blue method. Trypan blue dye was added to ASMCs detached and suspended in 1ΧPBS solution and live cells that did not absorb the dye were counted using a Neubauer slide.

### Data analysis

In ASMCs contractility studies each point represents the mean value of 5 cells, while in [^3^H]NMS binding and cell proliferation experiments each point was performed in triplicate. In immunofluorescence experiments the percentage of cells expressing the contractile proteins was estimated by counting the cells that were fluorescent, as well as the total cell number as indicated by DAPI staining. Image analysis of the images obtained from the Western blotting and the contractility studies was conducted with the use of MacBiotronics Image J programme for Light Microscopy (National Institutes of Health, Bethesda, MD, USA) and results were expressed as intensity values or as percentage of the initial cell area, respectively.

All data are expressed as means ± SEM and N refers to the number of independent experiments. Differences between means were analysed by Unpaired t-test with statistically significant differences between groups being determined by Mann-Whitney test. A comparison was considered significant when P < 0.05. The statistical analysis was performed using Graph Prism.

## Results

### The effect of muscarinic agonists, acetylcholine (ACh) or carbachol (CCh), on ASMC phenotype

**Figure 1:**
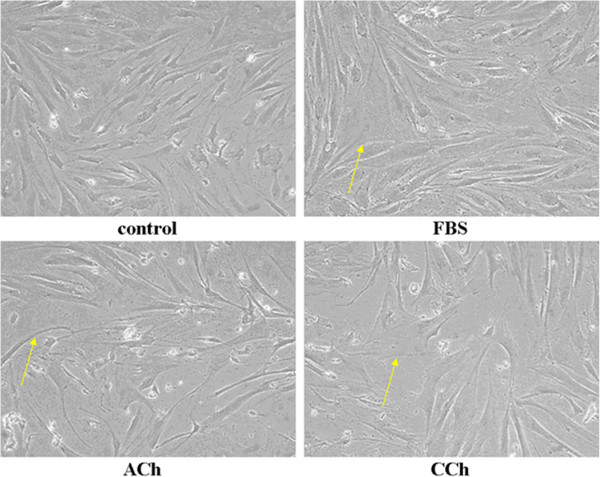
**Long-term treatment with muscarinic agonist affects ASMC morphology.** ASMCs were incubated for 30 days in serum free medium (control) or medium containing 10% FBS, ACh (10^-7^ M) or CCh (10^-9^ M) and their morphology was examined by optical microscopy (magnification × 20). The arrows are pointing at cells exhibiting synthetic phenotype.

Rabbit tracheal ASMCs were incubated in serum free medium for 24 h in order to get synchronised. ASMCs were then incubated in serum free medium, medium containing ACh (10^-7^ M), CCh (10^-9^ M) or 10% FBS for up to 30 days and their morphology was examined by phase contrast microscopy (Figure 1). ASMCs grown in the absence of FBS appeared elongated, while cells incubated in FBS-containing medium appeared flat. ASMCs incubated with ACh or CCh comprised a mixed population; part of which appeared flat, phenotypically similar to ASMCs incubated in the presence of FBS (indicated by arrows), while the rest maintained the elongated phenotype of cells incubated in serum free medium. The number of cells undergoing this morphological switch increased as time of incubation with ACh or CCh increased (data not shown).

**Figure 2:**
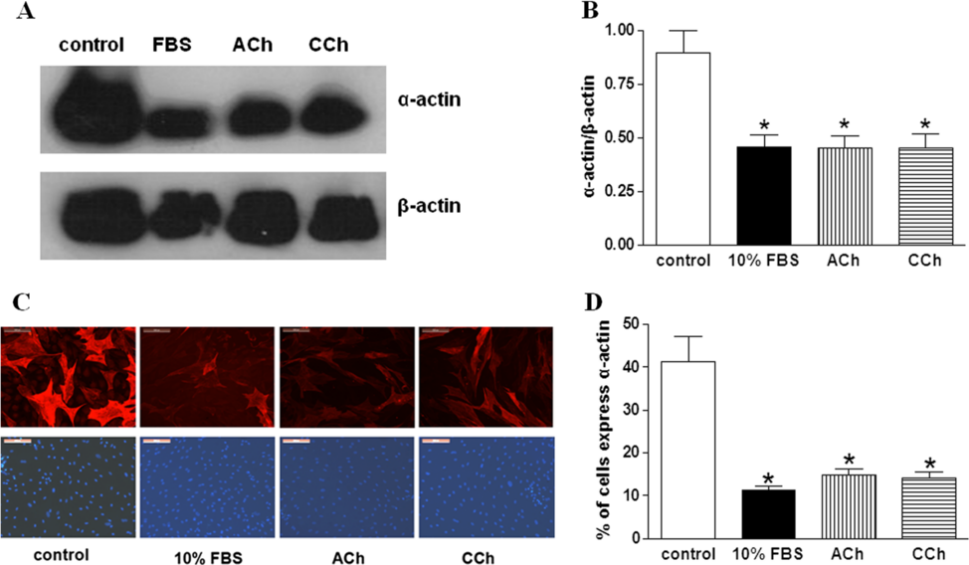
**Muscarinic agonists reduce the expression of smooth muscle α-actin in ASMCs. A**. Western blot analysis with anti-α-actin and anti-β-actin antibodies in total protein extracts from ASMCs incubated for 15 days in serum free medium (control) or in medium containing 10% FBS, ACh (10^-5^ M) or CCh (3 × 10^-7^ M). **B**. Quantification of α-actin expression normalized to the expression of β-actin presented in **A**. **C**. Specific α-actin expression by indirect immunofluorescence in ASMCs incubated for 15 days in serum free medium (control) or in medium containing 10% FBS, ACh (10^-5^ M) or CCh (3 × 10^-7^ M). Nuclei were stained with DAPI (lower panel). **D**. Counting of the percentage of rabbit tracheal smooth muscle cells which express α-actin in **C**. Ν = 4 independent experiments. *P < 0.05 compared with control.

**Figure 3:**
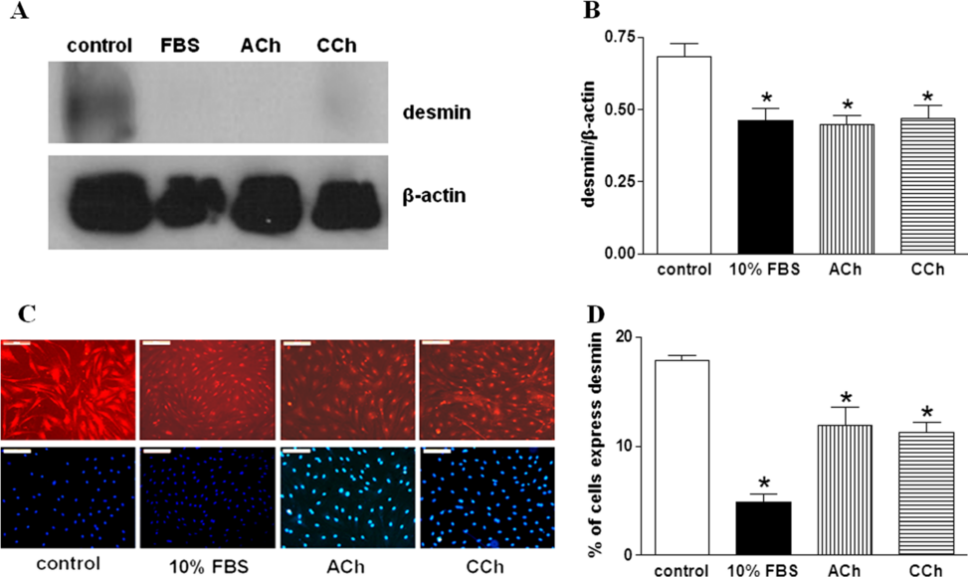
**Muscarinic agonists reduce the expression of contractile phenotype marker desmin in ASMCs. A**. Western blot analysis with anti-desmin and anti-β-actin antibodies in total protein extracts from ASMCs incubated for 15 days in serum free medium (control) or in medium containing 10% FBS, ACh (10^-5^ M) or CCh (3 × 10^-7^ M). **B**. Quantification of desmin expression normalized to the expression of β-actin presented in **A**. **C**. Specific desmin expression by indirect immunofluorescence in ASMCs incubated for 15 days in serum free medium (control) or in medium containing 10% FBS, ACh (10^-5^ M) or CCh (3 × 10^-7^ M). Nuclei were stained with DAPI (lower panel). **D**. Counting of the percentage of rabbit tracheal smooth muscle cells which express desmin in **C**. Ν = 4 independent experiments. *P < 0.05 compared with control.

**Figure 4:**
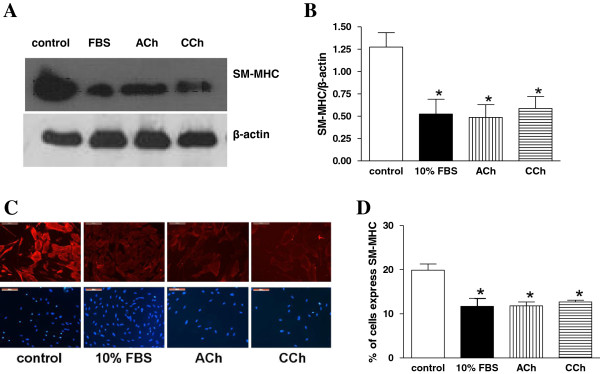
**Muscarinic agonists reduce the expression of contractile phenotype marker smooth muscle myosin heavy chain (SM-MHC) in ASMCs. A**. Western blot analysis with anti-SM-MHC and anti-β-actin antibodies in total protein extracts from ASMCs incubated for 15 days in serum free medium (control) or in medium containing 10% FBS, ACh (10^-5^ M) or CCh (3 × 10^-7^ M). **B**. Quantification of SM-MHC expression normalized to the expression of β-actin presented in **A**. **C**. Specific SM-MHC expression by indirect immunofluorescence in ASMCs incubated for 15 days in serum free medium (control) or in medium containing 10% FBS, ACh (10^-5^ M) or CCh (3 × 10^-7^ M). Nuclei were stained with DAPI (lower panel). **D**. Counting of the percentage of rabbit tracheal smooth muscle cells which express SM-MHC in **C**. Ν = 4 independent experiments. *P < 0.05 compared with control.

For further evaluation of the effect of muscarinic agonists on ASMCs morphology, 24 h-serum deprived ASMCs were cultured in serum-free medium or medium containing 10% FBS, ACh (10^-5^ M) or CCh (3 × 10^-7^ M) for up to 15 days and the expression of smooth muscle specific markers was studied. The mean or individual cell expression of smooth muscle specific proteins α-actin, desmin or SM-MHC, was analyzed by western blot analysis or indirect immunofluorescence, respectively. Incubation of ASMCs in the presence of ACh or CCh resulted in a decrease of individual cell and mean expression of α-actin (Figure 2), desmin (Figure 3) and SM-MHC (Figure 4), compared to ASMCs incubated in serum free medium. Moreover, this reduction in smooth muscle specific protein expression was similar to that of FBS.

### The effect of muscarinic agonists on muscarinic receptor expression

**Figure 5:**
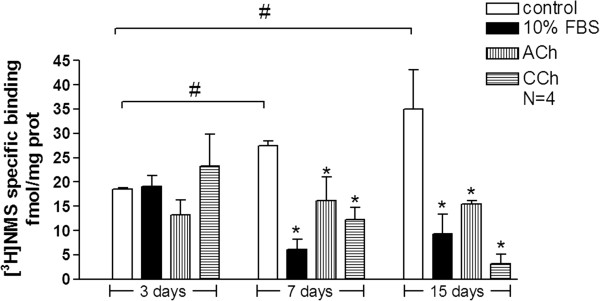
**The effect of muscarinic agonists ACh and CCh on N-methyl-[**^**3**^**H]scopolamine ([**^**3**^**H]NMS) specific binding.** Rabbit tracheal ASMCs were incubated for 3-15 days in serum free medium (control) or medium containing 10% FBS, ACh (10^-5^ M) or CCh (3 × 10^-7^ M). Specific binding was calculated using 5 nM of [^3^H]NMS in triplicate for each condition. ^#^P < 0.05 compared with cells serum starved for 3 days. *P < 0.05 compared with the respective control.

In order to determine the presence of M_3_ receptors, ASMCs were subjected to serum starvation for 24 h, and then incubated in serum free medium (control) or medium containing ACh (10^-5^ M), CCh (3 × 10^-7^ M) or 10% FBS for 3-15 days. Cell membrane fractions were isolated, equal amounts of protein (30 μg) were incubated with [^3^H]NMS (5 nM) for 80 min at room temperature in the presence or absence of 100 μM atropine and specific binding of [^3^H]NMS was calculated. Binding of [^3^H]NMS to ASMC membranes was similar in all samples after 3 days, but was significantly lower compared to respective control (P < 0.05) after 7 or 15 days of incubation in ACh, CCh or FBS (Figure 5). Interestingly, prolonged incubation in serum free medium, increased the binding of [^3^H]NMS to membranes of control cells incubated to 7 or 15 days compared to cells incubated for 3 days in the absence of FBS.

### The effect of muscarinic agonists on ASMCs contractility

**Figure 6:**
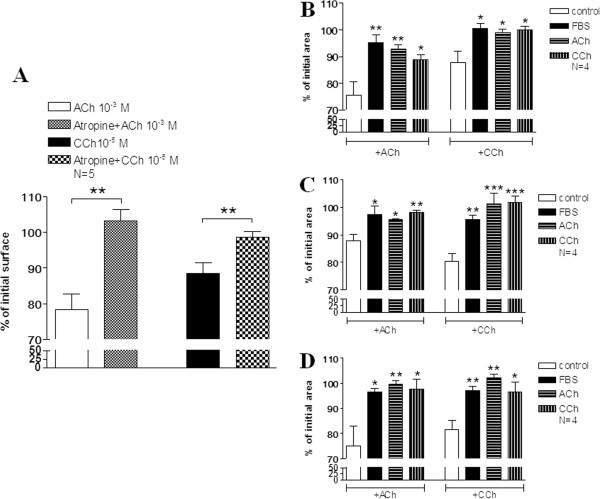
**The effect of 3-15 days of incubation with muscarinic agonists ACh and CCh on ASMC responsiveness. A**. Cell responsiveness to ACh (10^-3^ M) or CCh (10^-5^ M) in the presence or absence of atropine (10^-6^ M). **B-D**. ACh (10^-3^ M) and CCh (10^-5^ M)-induced contractions of rabbit tracheal smooth muscle cells cultured as indicated in serum free medium (control), medium containing 10% FBS, ACh (10^-5^ M) or CCh (3 × 10^-7^ M) for 3 **(B)**, 7 **(C)** or 15 **(D)** days. Cell responsiveness to ACh or CCh was estimated as a percentage of the area of cell measured 5 min after the addition of ACh or CCh in cell medium to the initial area before the addition of muscarinic agonist. *P < 0.05 and **P < 0.01 compared with the respective control.

We then measured the effect of prolonged incubation with ACh or CCh on ASMCs contractility. Therefore, ASMCs were grown to confluence in culture plates, serum starved for 24 h and maintained for 3-15 days in serum-free medium, or medium containing 10% FBS, ACh (10^-5^ M) or CCh (3 × 10^-7^ M). Then, the medium was removed, PBS was added, ASMCs were detached from the plate, the cell’s area was measured, cell contraction was provoked by the addition of ACh (10^-3^ M) or CCh (10^-5^ M) for 5 min and the cell’s area was measured again. Cell responsiveness to ACh or CCh was estimated as percentage of the single cell area measured before and after the addition of ACh or CCh. In control cells at day 0, the addition of ACh or CCh in the culture medium for 5 min decreased the cell surface to 78.3 ± 4.3% or 88.4 ± 3% of initial area respectively (Figure 6A). This effect of muscarinic agonists was specific, as it was abolished in the presence of atropine (10^-6^ M) (Figure 6A). ASMCs cultured in 10% FBS showed a decreased responsiveness to ACh or CCh (P < 0.05; Figure 6B-D) compared to serum starved cells (control). Similarly, the responsiveness to both muscarinic agonists of cells cultured for 3 to 15 days in medium containing 10^-5^ M ACh or 3×10^-7^ M CCh was significantly reduced compared to control cells (P < 0.05; Figure 6B-D).

### The effect of muscarinic agonists on ASMC proliferation

**Figure 7:**
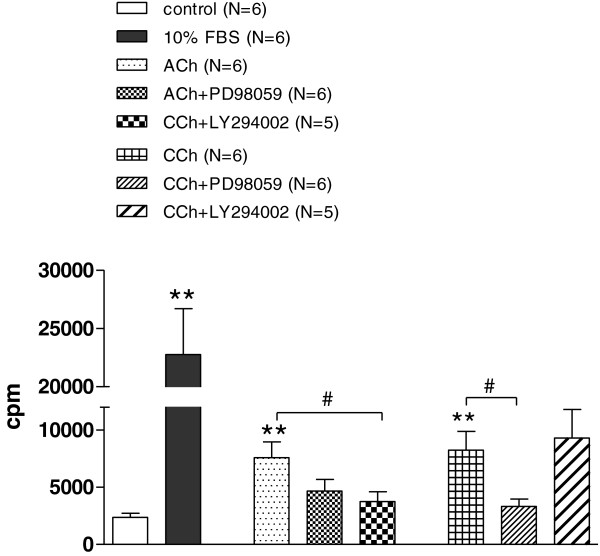
**Muscarinic agonists increase the methyl-[**^**3**^**H]thymidine incorporation in ASMCs.** Involvement of PI3K and MAPK signalling pathways. Rabbit tracheal ASMCs were serum starved for 24 h and then incubated with ACh (10^-7^ M) or CCh (10^-9^ M) alone and in the presence of the PI3K pathway inhibitor LY294002 (20 μΜ) or the MAPK pathway inhibitor PD98059 (100 μΜ). Methyl-[^3^H]thymidine incorporation was measured after 48 h of incubation with the muscarinic agonist. **P < 0.01 compared with control. ^#^P < 0.05 compared with the effect of muscarinic agonist alone on methyl-[^3^H]thymidine incorporation.

ASMCs were serum starved for 24 h and then treated with ACh or CCh in order to study the effect of the muscarinic agonists on ASMC proliferation by measuring methyl-[^3^H]thymidine incorporation and ASMC number. Both ACh (10^-7^ M) or CCh (10^-9^ M) caused a significant increase of methyl-[^3^H]thymidine incorporation after 48 h of incubation (P < 0.01, Figure 7). We next studied the involvement of the PI3K and MAPK signalling pathways in the induction of methyl-[^3^H]thymidine incorporation by ACh or CCh in rabbit tracheal ASMCs. The increase in methyl-[^3^H]thymidine incorporation caused by ACh, was lower in the presence of the PI3K and MAPK pathway inhibitors LY294002 and PD98059, respectively, although the effect of PD98059 was not statistically significant (Figure 7, middle group of bars). In contrast, only PD98059 but not LY294002 affected the increase in methyl-[^3^H]thymidine incorporation caused by CCh (Figure 7, right group of bars). Neither the PI3K inhibitor, LY294002 (20 μΜ) nor the inhibitor of MAPK pathway, PD98059 (100 μΜ) had an effect on methyl-[^3^H]thymidine incorporation in control cells, while they both decreased significantly methyl-[^3^H]thymidine incorporation, induced by the incubation of cells with 10% FBS for 48 h (data not shown).

**Figure 8:**
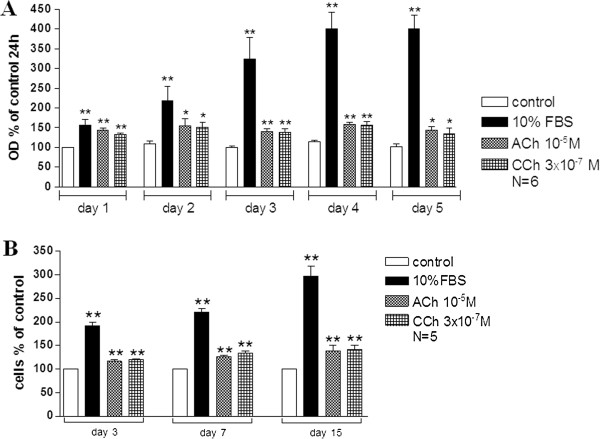
**Muscarinic agonists increase ASMCs number.** Rabbit tracheal ASMCs were incubated for 7 days in serum free medium before the addition of the muscarinic agonist in the culture medium for the indicated time. **A**. The effect of 1-5 days of incubation with 10^-5^ M ACh or 3 × 10^-7^ M CCh on rabbit tracheal smooth muscle cell number estimated with the MTT-Cell Titer 96® AQueous One Solution Assay (Promega) method. **B**. The effect of 3-15 days of incubation with 10^-5^ M ACh or 3 × 10^-7^ M CCh on rabbit tracheal smooth muscle cell number estimated with Trypan blue method. *P < 0.05 and **P < 0.01 compared with the respective control.

**Figure 9:**
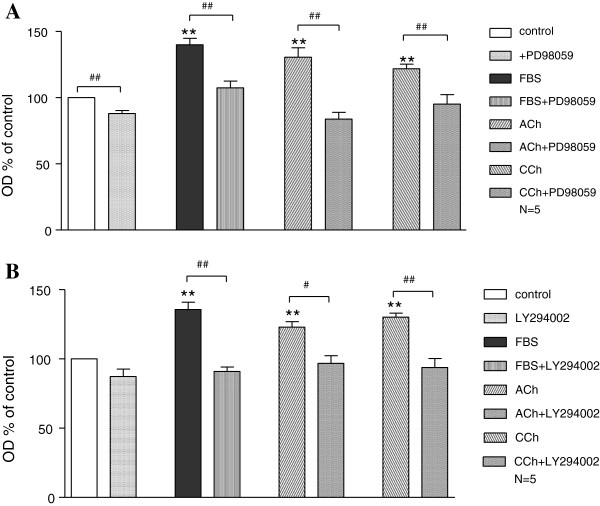
**The involvement of PI3K and MAPK signalling pathways on the muscarinic agonists induced proliferation of ASMCs.** Rabbit tracheal ASMCs were incubated for 7 days in serum free medium before the addition of either FBS (10%), ACh (10^-5^ M) or CCh (3 × 10^-7^ M) for 24 h with or without either PD98059 (100 μM) in **A** or LY294002 (20 μM) in **B**. **P < 0.01 compared with control. ^#^P < 0.05 and ^##^P < 0.01 compared with the effect of control, 10% FBS or muscarinic agonist alone on cell number.

Incubation of ASMCs with ACh (10^-5^ M) or CCh (3 × 10^-7^ M) under the same conditions (24 h serum starvation, incubation with ACh or CCh for 72 h) did not change rabbit tracheal ASMC number, as estimated using Cell Titer 96 AQueous One Solution Assay (Promega) method (data not shown). Previous reports have shown that serum deprivation increases the percentage of cells that express the “contractile phenotype” in ASMC culture [19] and the expression of M_3_ muscarinic receptors [11]. Moreover, as shown in Figure 5, incubation in serum free medium for 7 days increases binding of [^3^H]NMS to ASMC membranes. Thus it is possible that the lack of cell number increase by ACh or CCh, is caused by the absence of a potent number of M_3_ muscarinic receptors on the cytoplasmic membrane of ASMCs that were incubated for 24 h in serum free medium, before the addition of the muscarinic agonists. We, therefore, tested the effect of ACh or CCh on the proliferation of rabbit tracheal ASMCs that have been subjected to prolonged serum starvation and performed a time course experiment. Rabbit tracheal ASMCs were incubated for 7 days in serum free medium and then ACh (10^-5^ M) or CCh (3 × 10^-7^ M) was added to the culture medium for 1 to 15 days. Cell number was estimated with MTT-Cell Titer 96® AQueous One Solution Assay (Figure 8A) and Trypan blue method (Figure 8B). Both methological approaches revealed that ASMC number increased significantly compared to control after 1 day of incubation with ACh and CCh, while no further increase was observed after up to 15 days cell incubation with the muscarinic agonists (Figure 8). The mitogenic effect of ACh and CCh on rabbit tracheal ASMC number was significantly (P < 0.05) reduced by the MAPK inhibitor PD98059 (100 μΜ) (Figure 9A) as well as by the PI3K pathways inhibitor LY294002 (20 μΜ) (Figure 9B).

## Discussion

In airway smooth muscle, muscarinic agonists may activate signalling pathways such as the Rho kinase, PI3K or MAPK pathways [2]. Since the MAPK pathway is involved in modulation of ASMCs toward a less contractile phenotype [6,7,10], we hypothesized that muscarinic agonists could affect ASMC phenotype. Indeed, we found that rabbit tracheal ASMCs subjected to prolonged (30 days) incubation with ACh or CCh switched to a proliferative phenotype, i.e. cells appeared flat, similar to that observed in ASMCs cultured in the presence of 10% FBS. Accordingly, indirect immunofluoresence, as well as western blot analysis experiments demonstrated that this “induction” of a proliferative phenotype was accompanied by a significant decrease in the expression of the contractile phenotype markers α-actin, SM-MHC and desmin. To our knowledge, this is the first study reporting that muscarinic agonists induce the shift of ASMCs toward a proliferative phenotype. Our results are in agreement with a previous study by Gosens et al. [20] who showed that prolonged treatment with the muscarinic agonist methacholine of bovine tracheal smooth muscle strips in organ-cultures diminished the expression of myosin and actin.

Furthermore, there are studies indicating that the switch of ASMC phenotype from proliferative to contractile caused by incubation in the absence of FBS is accompanied by an increase in M_3_ expression [11]. Accordingly, our results demonstrate that the ACh or CCh induced shift of the cell phenotype towards a proliferative one, was followed by a decrease of the M_3_ receptors density, as shown by decreased [^3^H]NMS binding in cells incubated with ACh or CCh for 7-15 days. Evidence suggests that agonists may induce phosphorylation and internalization of muscarinic receptors [21]. In our study, the down regulation of M_3_ receptors of ASMCs was accompanied by decreased responsiveness of isolated cells, incubated for 3-15 days with muscarinic agonists, to ACh or CCh, due probably also by the decrease of smooth muscle protein expression. These results are in agreement with results obtained from contractility studies demonstrated that long-term methacholine pretreatment of bovine tracheal smooth muscle strips decreased maximal contraction and sensitivity to methacholine [20]. In vivo studies revealed increased cholinergic tone in asthma [22] and clinical studies showed that the addition of anti-cholinergic drugs to the corticosteroid or long-acting β₂-agonist treatment is beneficial for asthmatic patients [23,24]. On the other hand in vitro studies performed with bronchial smooth muscle from asthmatic patients showed that the altered airway responsiveness seen in asthmatic patients is not reflected in airway smooth muscle sensitivity in vitro and that the sensitivity to carbachol was significantly reduced in tissue from asthmatic patients [25,26].

Even though a more proliferative phenotype was induced by muscarinic agonists, this phenotype is not necessarily associated to increased proliferative capacity, since each phenotype has different expression of proteins, as well as receptors that affect the activation of signalling pathways that are involved in the proliferation process. We studied the proliferative effect of muscarinic agonists ACh and CCh on ASMCs that were serum starved for 24 h or 7 days. According to our results and previous studies [5], 24 h serum starved cells express mainly the proliferative ASMC phenotype while 7 days serum starved cells express an increased percentage of ASMCs with contractile phenotype. We found that ACh and CCh increased [^3^H]thymidine incorporation in rabbit tracheal ASMCs, serum starved for 24 h before muscarinic agonists addition (Figure 7), but did not affect cell number (data not shown). On the other hand when cells grew in serum free medium for 7 days prior to the addition of ACh and CCh, both muscarinic agonists significantly increased cell number (Figures 8 and 9). This effect was transient since no further increase was observed after 24 h (2-15 days incubation with muscarinic agonists). These results suggest that the mitogenic effect of muscarinic agonists depends on the ASMC phenotype. Although, in airways, muscarinic agonists have a proliferative effect on fibroblasts [27,28] until now available data demonstrate that stimulation of muscarinic receptors is not sufficient to induce ASMC proliferation, but the synergetic muscarinic receptor stimulation with growth factors may cause an induction of mitogenesis [29]. However, our results demonstrate that the mitogenic effect of muscarinic agonists may depend on ASMC phenotype, since it seems reasonable that the decrease of M_3_ receptor expression in cells cultured in the presence of FBS, ACh or CCh is responsible for the weak mitogenic effect of muscarinic agonists.

It has been reported that the stimulation of muscarinic receptors in ASMCs activates the PI3K or MAPK pathways [3]. However, the PI3K pathway inhibitor LY294002 reduced [^3^H]thymidine incorporation induced by ACh, while MAPK pathway inhibitor PD98059 reduced [^3^H]thymidine incorporation induced by CCh. These results suggest that whereas the stimulation of muscarinic receptors may cause the [^3^H]thymidine incorporation in rabbit tracheal ASMCs via either PI3K or MAPK pathways, these pathways are activated differentially by ACh and CCh. Both ACh and CCh are agonists of all muscarinic and also nicotinic receptors. Although the main difference between ACh and CCh is their resistance to the cholinesterase activity, some data suggest that acetylcholine and carbachol interact with different muscarinic receptors [30,31]. However both LY294002 and PD98059 abolished the effect of ACh and CCh on ASMC number suggesting that although ACh and CCh may activate PI3K or MAPK pathways differently, the activation of both pathways is required for ASMC proliferation.

## Conclusions

In conclusion, muscarinic receptor stimulation may shift ASMC phenotype to “proliferative”, reduce ASMC responsiveness and have a transient mitogenic effect, via MAPK and PI3K pathway signalling, depending on the ASMC phenotype. These results may have clinical significance given that in chronic inflammatory airway diseases increased acetylcholine release is observed in a periodical pattern, for example during asthma attacks [2]. Therefore, the presence of acetylcholine in the airway can lead to alteration in the ASMC phenotype that makes them more prone to be affected by growth or inflammatory factors present in the airway in such diseases, and lead to increased proliferation rate. Both the phenotype shift and the increased proliferation can lead to the ASMC hypertrophy and hyperplasia, which are characteristics of airway remodeling. Furthermore, there are studies that propose that muscarinic agonists have an immediate effect on airway tone, since they can increase the contractility of isolated ovine tracheal strips [2,32-34]. This acute effect of muscarinic agonists on airways, accompanied by changes in ASMC phenotype and induction of cell proliferation under prolonged incubation, indicates the significance of muscarinic agonists in the physiology and pathophysiology of airway function.

## Abbreviations

ACh: Acetylcholine; ASMC: Airway smooth muscle cell; BSA: Bovine serum albumin; CCh: Carbachol; COPD: Chronic obstructive pulmonary disease; DMEM/F12: Dulbecco’s modified Eagle’s medium/Ham/F12; FBS: Fetal bovine serum; MAPK: Mitogen activated protein kinases; SM-MHC: Smooth Muscle Myosin heavy chain; PI3K: Phosphatidyl inositol kinase.

## Competing interests

The authors declare that this manuscript has been financed by Boehringer Ingelheim Pharma GmbH & Co, KG, Biberach an der Riss, Germany. Specifically, Boehringer Ingelheim Pharma GmbH & Co has funded the experimental procedure, paid the salary of the researcher and is financing the article-processing charge. The authors do not hold any stocks or shares in an organization that may in any way gain or lose financially from the publication of this manuscript, either now or in the future. The authors are not currently applying for any patents relating to the content of the manuscript, or have not received reimbursements, fees, funding, or salary from an organization that holds or has applied for patents relating to the content of the manuscript. The authors have no other financial or non-financial (political, personal, religious, academic, ideological, intellectual, commercial or any other) competing interests.

## Authors’ contributions

RS has been involved in acquisition, analysis of data and interpretation. EP has been involved in data analysis and interpretation and participated in the design of the study. AV has been involved in data acquisition, analysis and interpretation IM has been involved in data acquisition and analysis. PAM conceived of the study and participated in its design. KG conceived of the study and participated in its design. AH has designed and coordinated the study and participated in data analysis and interpretation. All authors have read, commented on and approved of the manuscript.

## Pre-publication history

The pre-publication history for this paper can be accessed here:

http://www.biomedcentral.com/1471-2466/14/39/prepub
